# Women medical missionaries in Kashmir from the late 1800s to the early 1900s

**DOI:** 10.1186/s12939-026-02901-3

**Published:** 2026-06-05

**Authors:** Hilal Ahmad Tantray

**Affiliations:** Department of History and Culture, Jamia Millia Islamia, New Delhi, India

**Keywords:** Zenana, Medical, Missionaries, Kashmir, Purdah

## Abstract

Female medical missionaries significantly contributed to the advancement of healthcare practices in colonial Kashmir, especially in women’s healthcare, nursing, midwifery, and medical outreach. This article analyzes the role of female medical missionaries in the development of institutional healthcare in Kashmir from the late nineteenth to the early twentieth centuries. It inquires how missionary women navigated local social and cultural obstacles, including gender norms and purdah regulations, to enhance access to medical care for Kashmiri women. The study examines the convergence of medicine, gender, and colonial action in the region by utilizing missionary records, official reports, travelers’ accounts, and secondary historical sources. The research contends that female medical missionaries not only introduced Western medical methods but also influenced new modalities of public health involvement through dispensaries, prenatal care, nursing services, and medical education. The study contextualizes its findings within the extensive history of medicine in Kashmir, emphasizing the gendered aspects of healthcare evolution and the frequently neglected role of women in the region’s medical narrative.

## Methodology

This study uses a historical and qualitative research approach to investigate the role of female medical missionaries in Kashmir in the late nineteenth and early twentieth centuries. The study takes an interdisciplinary approach, combining social history of medicine, feminist historiography, and postcolonial analysis, to analyze how missionary medicine intersected with gender, colonial government, religion, and indigenous society during Dogra reign.

The study draws on both primary and secondary sources. Primary sources include missionary memoirs, autobiographies, institutional reports, trip narratives, church documents, and contemporary publications about missionary activities and medical care in Kashmir. Key materials consulted include Ernest F. Neve’s *Beyond the Pir Panjal*, E. M. Tonge’s *Fanny Jane Butler: Pioneer Medical Missionary*,* Seed Time in Kashmir*,* Irene Petrie’s missionary biography*, and Tyndale Biscoe’s publications. These texts contain useful information about hospital administration, women’s healthcare, zenana work, medical evangelism, and missionary relations with Kashmiri society. Regional histories and contemporary reports, such as the works of G. M. D. Sufi and other Kashmir-related historical publications, have also been used to contextualize the period’s social, cultural, and medical environment.

The study also looks at recent scholarship on colonial medicine, missionary enterprises, and gender history, specifically the works of David Hardiman, Maina Chawla Singh, Christoffer Grundmann, Mridula Ramanna, and Kamlesh Mohan. These studies give the broad historiographical framework required to situate Kashmir within larger discussions about colonial medicine, medical pluralism, women’s healthcare, and missionary engagement in South Asia.

The research takes a critical approach to missionary-authored sources rather than accepting them as impartial historical records. Missionary memoirs and institutional reports were typically affected by evangelical, imperial, and fundraising goals, and so described Kashmiri culture, indigenous medical practitioners, and local customs through a civilizing and reformist lens. Purdah, indigenous healing traditions, and women’s healthcare practices must all be viewed as ideologically produced narratives interwoven in colonial ideologies of progress, morality, and modernity. Drawing on postcolonial scholarship and feminist historiography, the paper investigates how missionary medicine served as a humanitarian venture, a gendered intervention, and a component of larger colonial knowledge systems.

To address these constraints, missionary texts have been compared to regional histories, studies on indigenous medicine, colonial administrative documents, and contemporary historical scholarship. This comparative method enables the study to transcend beyond celebratory missionary narratives, resulting in a more balanced assessment of women medical missionaries and their impact on healthcare, gender relations, and medical modernity in Kashmir.

## Introduction

This study argues that female medical missions in Kashmir were not just charitable efforts; they were also a strategic tool for spreading the gospel, with medical practice serving as both healing and conversion. As one missionary formulation stated, “A female medical mission may be defined as the practice of medicine by a lady, for the purpose not merely of curing, but with the primary aim of the Christian missionary enterprise, which had always been to convert” [1]. Thus, medicine operated as both therapeutic intervention and missionary methodology. In India, Medical Missionary work began in the early nineteenth century. Medical missionaries played a prominent role in healthcare not only in India but also in Kashmir. An Anglican bishopric of Calcutta was established by the British Parliament in 1813, and missionaries were officially licensed to enter the territories of the East India Company [2]. This study highlights that missionary medicine’s feminization represented a substantial shift from colonial medicine’s male-dominated practice. British women entered the mission field as wives. In the words of St. John Rivers to Jane Eyre, a missionary wife was to be her husband’s help meet and fellow laborer, a shining example to the heathen of Christian femininity and domesticity Chatterton [3].

Dr. Elmslie quoted “India is not now an entire stranger to female medical missions. In the provinces of Northern India female medical missionaries are already at, lessening pain, saving life, and training native Christian women for the same end” [1].

Women medical missionaries played a significant role in challenging patriarchal norms and advancing healthcare in colonial India, particularly in Kashmir. Despite facing gender based discrimination in medical education and practice [4] these women established important medical institutions and pioneered research. They demonstrated remarkable financial and administrative skills, transforming modest ventures into state institutions through strategic alliances and resource management. The struggle for medical education by Indian women during the colonial period had a profound impact on social reform movements and women’s empowerment across caste, class, and regional boundaries. By establishing zenana dispensaries, maternity services, and female medical wards, women medical missionaries helped Kashmir’s female healthcare system become more institutionalized. In settings where women were unable to engage with male doctors due to purdah regulations, their work increased their access to biomedical care [4]. A more efficient method of missionary work was advocated for by Doctor William Elmslie, who is widely regarded as the pioneer of medical mission in Kashmir. He quoted “*Is there no other key but that of Education with which to open the door to the inner social life of India? We think there certainly is one other such key*,* and that key is female medical missions…. the practice of medicine by a lady*,* for the purpose*,* not merely of curing*,* but Christianizing her patients… This is a key that may be said to fit every lock…. She would find an entrance where the educational missions would find the door closed*” [1].

His call for the development of female medical missions was based on the ground that in India “the state of things is peculiar and exceptional which not only warrants, but demands peculiar and exceptional measures” (Elmslie and Thompson 1875, 181) [1]. This article emphasizes that female medical missions were intentionally structured to infiltrate zenana environments and reach the inner domestic realm, thus transforming gendered medical authority.

To analyze the women medical missionaries in Kashmir, one must investigate the intricate dynamics. Prioritize culture; women acceptance in medicine varies according to local gender norms and cultural expectations. Subsequently, evaluate the gender dynamics inside missions; female missionaries can both contest power systems and perpetuate inequity.

Health disparities among Kashmiri women exacerbate the situation, female medical missionaries may endeavor to tackle certain health issues; nevertheless, they must evaluate if their approaches are appropriate for the local context or neglect critical concerns. The history of Kashmiri missionaries is very significant. This necessitates comprehending colonial consequences and the influence of past actions on contemporary perceptions of international medical help. David Hardiman argues that the missionaries’ “strong focus on women was one of the most radical components of mission medicine, distinguishing it sharply from the medical practice of the colonial state” [5].

The women of the valley are fortunate in having lady doctors in charge of the Zenana Hospitals who have never spared themselves in their solicitude for their suffering sisters. In Srinagar the women have been blessed in having amongst them English women who have for years given of their best to relieve them in their times of sickness and distress [6]. Healthcare and religion can help or hurt female missionaries, altering their acceptance and efficacy. Ethics are also important. Women missionaries typically struggle with autonomy and foreign values, posing ethical concerns. Finally, the impact of women medical missionaries on local healthcare systems must be assessed. They can help increase capacity, but they must be sustainable and respectful of local practices. Women medical missionaries in Kashmir have a complex historical background that is impacted by multiple important variables. Significant colonial expansion, notably by Britain in India, occurred during the 1800s and 1900s, paving the way for missionary activity. Missionary activity, especially in healthcare, was shaped by the growing involvement of women in social reform during this time in the West. Thus, this study contends that the gendered accessibility of healthcare in the Valley, especially in Zenana hospitals, was changed by female medical missionaries [7].

Medical institutions also rose in prominence between 1850 and 1900, reflecting a growing belief that the Gospel was not only a gift for the transformation of the spirit, but also for the body. Oddie [8], Modern medical science and the rise of Western medicine have drawn attention to the differences between these two approaches. As part of a larger civilizing mission connected to their religious beliefs, missionaries frequently sought to promote these behaviors. Women in Kashmir also had restricted access to healthcare and education during this time, adding to the complexity of the social climate. The intersection of female missionaries work with local reform movements presented women in the region with both obstacles and opportunity. To summarize, the complex interplay of cultural, social, and political elements in shaping the experiences and local setting of the women medical missionaries in Kashmir is essential to comprehending their missionary work.

Women medical missionaries were crucial to Kashmiri healthcare in the late 1800s and early 1900s. These motivated people, often missionaries, went to the culturally rich region to address healthcare disparities and provide medical treatment. These women handled Kashmir’s difficult terrain with medical competence and compassion, improving healthcare and leaving lasting imprints on their communities. Traditional practices dominated Kashmir’s health scene until [9] medical missionaries arrived in the 1800s and 1900s. Ayurveda and herbal medicine, used by women healers, were important to the indigenous populace. Healing with herbs and spirituality, these healers were vital to their communities. Spiritual and supernatural forces shaped cultural health perceptions. Many illnesses were seen as symptoms of imbalances or external influences, prompting healing rituals and community practices. The region had few hospitals, clinics, and medical schools, limiting access to formal medical care.

As Christian missionaries arrived in Kashmir, some were female doctors by profession, receiving significant support from other female medical missionaries. Additionally, there were missionary couples where the wives, although not professionals themselves, played a crucial role in assisting with medical care. These women, educated about the medical system through their husbands, became integral to healthcare provision in Kashmir. Initially, in the late 19th century, only male medical missionaries operated in Kashmir, posing communication challenges for female patients unable to articulate their illnesses to male doctors. However, as time progressed, with the emergence of female medical missionaries, these patients gained the opportunity to express their conditions more effectively to female doctors, thereby enhancing the quality of medical assistance provided. This does not surprise most academics who label missionary women as “conservative” [10]. However, some historians have stressed that missionary women contributed, perhaps unwittingly, to the development of Indian and other Asian feminists and nationalists by establishing boarding schools and opportunities for more advanced education for girls and young women.

Although the beginning of female medical care was initiated by Mrs Clark in Kashmir in the year 1864, long before the establishment of female medical mission in Jammu and Kashmir. She opened first informal female dispensary for women in the State [1]. Being female, most of the patients usually lower caste women, visited her for their problems. This was because caste women mostly lived in purdah and also the idea of getting polluted by the touch of a Christian lady never allowed them to visit missionary dispensary. In a letter to Mr. Coldstream, Mr. Clark gave the description of work of Mrs. Clark’s dispensary that she had to work daily for three hours and sometimes had to attend eighty patients [1].

This was the beginning of any kind of modern female health care in the State. Beyond their medical achievements, women medical missionaries in Kashmir in the 19th and 20th centuries changed society’s view of women’s health and education. These ladies challenged traditional norms by providing needed healthcare and advocating for women and girls education in local communities. These tensions reveal that missionary medicine functioned within complex social hierarchies rather than operating as a universally accepted reformist force. Missionary medical endeavors indirectly stimulated discussions around female education, maternal healthcare, sanitation, and women’s involvement in nursing and midwifery training. These advances, however restricted in scope, intertwined with wider colonial and indigenous discourses regarding women’s welfare and public health reform. In a region where women were traditionally domestic, this was noteworthy. Their work also created medical facilities that gave local women a unique chance to study healthcare, nurturing a new generation of female healthcare workers. Ghosh D notes that such attempts created a unique canon of juvenile writing, expressing a growing sense of identity and agency in colonized situations, which is reflected in these missionaries legacy. In Kashmir, the effects of female medical missionaries were varied and complicated. While missionary organizations brought novel medicinal treatments to women, their operations continued to be entwined with local patterns of negotiation and resistance, evangelical goals, and colonial structures. Women medical missionaries became the only recognized formal healthcare providers for high-caste and Muslim women in Kashmir, where purdah was strictly maintained and local female healers (*Warren/dais*) were shunned. This essay contends that their efforts left behind a paradoxical legacy that was both emancipatory, evangelical, and supportive of colonial structures.

## Health system of women before arrival of missionary

The people of the state believed that a disease was caused by the will of God. Therefore, instead of visiting a doctor when they discovered any illness, they usually sought remedy from their religious men, the priests [11]. Usually, the priests handed them a sheet of paper on which the Brahman priest scribbled “Shiva” and the Muslim Mullah penned “Allah”. The patients drank it down with water, generally contaminated, filthy, and harmful. Under these conditions, the death rate could not but be rather heavy. Regarding small-pox, the Hindus were more delighted that their goddess Sheitala, to whom the illness was ascribed, had opted to pay visit to their modest homes. They therefore fiercely protested to vaccination [11].

Wearing charms or amulets was another common way to treat them. These charms were usually small pieces of paper with a verse from the Quran or a picture of a god on them. A piece of paper was rolled up and sewn into a cloth or leather cover. It was also put in a small copper case that was about one or two square inches. People with illnesses had this kind of charm put on the part of their body that was sick [12]. But as western education expanded, people’s superstitions and ignorance started to wiggle free. People came to see that an illness was brought on by germs and, so, could be either prevented or healed with appropriate therapy. People started to adopt the western style of medicine progressively after this enlightenment [11].

Female seclusion is complete confinement behind purdah. Ancient India didn’t follow purdah. Foreign invasions led to the creation of purdah and Jauhar to protect noble and royal ladies. In Kashmir, high class Rajput and Muslim women scrupulously observed purdah and were ‘purdanashin’ [11]. When it comes to women health care, the purdah became a significant obstacle that created a massive difficulty. It also made it difficult for women to receive adequate medical care. It became common for women to keep their issues hidden and to avoid going to medical professionals, which led to a neglect of their health situation. In the event that a woman became ill, she never went to a male physician, and she did not have access to female physicians during that time period. As a result, women opted to pass away rather than receive medical treatment.

Before western medicine and doctors from the west arrived in Jammu and Kashmir, the state’s *Hakims* and *Vaids* took care of the people’s health needs. A distinct group of female assistants was recognized in different parts of the world for gynaecological and women specific concerns. In Kashmir region, they were called ‘*Warren*’. Typically, *dais* belonged to lower castes, such as doms, chamars, or other scheduled castes, as well as lower caste Muslim women, and their occupation was despised [13]. Women who served as dais were typically born into the category, as the status passed from mother to daughter; it was not the type of work anyone wanted to do as Supriya Guha explains, it was “ascriptive rather than aspirational” work. Finally, as medical women often complained, even in areas where there were fully-trained midwives available, many Indian women preferred dais to “skilled” help, so equipping the dais with “skill” seemed the best planto provide competent aid to women in labor. They lacked formal training and either learned these abilities on the job or inherited them from their ancestors or via personal experience. *Dais/Warren* were the only people who could care for women when they had ailments that were specific to them. Midwifery became “the backbone of district medical work” by the 1930s [14]. Women occasionally sought advice from male physicians for general illnesses, but only through the intervention of male family members [15]. On behalf of the sick woman, the male family member went to see a male doctor and explained her symptoms; the male doctor then continued treating her via a messenger. ‘*Messenger Health Care’* or ‘*Carrier Health Care*’ was in vogue during this time [16]. Through a veil that served as a screen, a male doctor was occasionally permitted to view a female patient. Through the curtain dividing them, the doctor could feel the pulse of the women sitting on one side and the doctor on the other [17].

Western medical professionals were very critical of how dais worked. Katheline Vaughan said that dais cleaned their hands on the mud floor before starting their work by examining a patient [13]. They didn’t care about cleanliness or health and used old, broken tools to do their work. They also didn’t think about sterilization because they didn’t know enough about germ theory. People thought that the rough ways they used to give birth were to blame for the high death rates among mothers and babies [13]. Dr. Elmslie said that the local nurses didn’t know what they were doing, got in the way of doing their jobs, and were generally bad people. After missionaries set up health care facilities for women, the Dias were thought to know nothing about the physiology or process of childbirth. People thought that their unpleasant actions during labor and the dirty conditions of the place where the birth took place were the main reasons why both the mother and the baby died [1].

In the nineteenth century, the medical care available to women in Kashmir was a mix of indigenous practices, home remedies, and limited professional medical services. Women’s healthcare in Kashmir was a mix of traditional practices, home cures, and limited professional medical services. The primary healthcare providers were Hakims, who practiced Unani medicine, which incorporated herbal remedies, dietary guidelines, and natural medicines. Ayurveda also played a role, focusing on balancing the body’s energies through diet and lifestyle modifications, as well as offering specialized cures for women’s health difficulties. Home medicines were prevalent, with locally accessible herbs such as saffron, garlic, and turmeric. Traditional midwives, known as dais, aided in childbirth by using herbal remedies and warm oil massages to ease labor and promote postpartum healing [18].

However, maternal health care was minimal, resulting in high mother and newborn death rates. While physical labor and nutrition were the main ways to control general health concerns, frequent ailments like respiratory infections and tuberculosis were also common. The first hospitals appeared later in the century, primarily serving metropolitan areas, and the health infrastructure was very limited. Western medical procedures were gradually brought by British colonial control, but their impact on the rural populace was limited and their adoption was sluggish. Traditional approaches predominated overall, offering basic treatment but falling short in successfully addressing major health conditions.

Indigenous people’s reception to missionaries was complicated. Others welcomed new medical understanding and enjoyed emergency healthcare. However, suspicion and resistance abounded. Missionaries were seen as colonial agents, therefore natives distrusted foreign intrusions. Traditional healers felt intimidated by Western medicine, causing tensions. Local communities sometimes combined Western and traditional medicine to create a more inclusive healthcare approach [19]. Missionaries sometimes struggled to acquire confidence and acceptance due to local beliefs and customs. Medical missionaries met important health needs in Kashmir, but their interventions provoked a variety of responses that mirrored the complexity of cultural interchange in a colonial framework.

Although missionary discourse sometimes portrayed medical labor as a means of evangelism, the evidence does not indicate substantial rates of religious conversion among Kashmiri patients, particularly women treated in zenana missions. Instead than generating quantifiable Christian affiliation, missionary medicine mostly served as a vehicle for social access and cultural interaction. The majority of patients seem to have selectively adopted biological treatment for therapeutic reasons while maintaining their religious beliefs and cultural customs. Consequently, medicine in colonial Kashmir ought to be perceived not merely as an effective tool for conversion, but rather as a contentious arena of engagement, negotiation, and constrained missionary impact.

In order to influence women and ultimately convert them to Christianity, CEZMS specialized in sending female missionaries to nations, cities, villages, and communities that had been colonized by the British. The female missionaries approach included zenana visits, nursing and medical care, and classroom instruction [18]. One reason is that the area’s natural beauty served as a source of inspiration for female medical missionaries, who began traveling here. They were drawn to this location in large part by the beautiful scenery and calm atmosphere. The weather is an additional factor. It was Kashmir’s pleasant weather that drew these missionaries to the region. Kashmir has far milder and more pleasant summers than other parts of the country where they can get very scorching. They found it to be a popular destination because of its favorable climate.

## Indigenous women’s agency

The presence of women missionaries was crucial in negotiating cultural barriers, particularly the practice of *purdah* (seclusion), which restricted male physicians from accessing women within Kashmiri households [20]. Female missionaries and medical practitioners were therefore uniquely positioned to enter domestic spaces and provide healthcare to women and children who often remained beyond the reach of formal medical institutions. Colonial and missionary writings frequently portrayed Kashmiri women as isolated, medically uninformed, and dependent upon missionary assistance. However, such representations reflected the ideological assumptions of missionary discourse more than the lived realities of Kashmiri society. In practice, Kashmiri women exercised significant agency in negotiating the terms of treatment, selectively accepting biomedical procedures while simultaneously preserving their cultural and religious identities. They were not merely passive recipients of missionary medicine but active participants in shaping their own healthcare choices.

The missionary movement also created limited avenues for women’s participation in healthcare and social outreach. The Church of England Zenana Missionary Society, founded in 1880, began its activities in Kashmir in 1886. Its first representative, Mrs. Rallia Ram, the daughter of a local Christian priest, gained access to several zenanas as an honorary worker [21]. In particular, zenana medicine opened new channels of interaction between missionary women and Kashmiri households, allowing female missionaries to reach women who remained inaccessible to male doctors because of purdah restrictions. However, the relationship between missionary medicine and Kashmiri women was not simply one of religious conversion or passive acceptance. Many women visited missionary hospitals and dispensaries primarily for practical therapeutic reasons, especially in matters related to childbirth, gynecological illness, and child healthcare. Acceptance of Western medicine therefore did not necessarily imply acceptance of Christianity. Despite adhering to Islamic social norms and local healing traditions, Kashmiri women often selectively utilized missionary medical services while maintaining their own cultural and religious identities.

It is also necessary to critically examine missionary criticisms of indigenous midwives (dais), who were frequently described as “ignorant” or “unclean.” Although Fanny Jane Butler highlighted important public health concerns in Kashmir through her medical work, her writings also reflected broader colonial attitudes toward indigenous society and healing practices. In one account, when Butler asked Kashmiri women why they paid little attention to cleanliness, they reportedly replied, “We have been so oppressed, we don’t care to be clean” [22]. Such descriptions portrayed Kashmiri society as socially backward and reinforced colonial medical assumptions that sought to discredit indigenous systems of knowledge in order to legitimize missionary intervention. However, dais continued to occupy an important social and cultural position within Kashmiri society because of their accessibility, community trust, and practical experience in childbirth. Rather than disappearing with the arrival of missionary medicine, indigenous practitioners often coexisted alongside biomedical institutions within a broader framework of medical plurality.

Although chances for female medical training, such as nursing and midwifery training, remained limited and poorly documented in Kashmir compared to other parts of colonial India, there is also scant evidence that missionary organizations created tiny channels for such training. Even though this procedure was still limited by class, religion, and purdah customs, missionary hospitals let women gradually into public medical settings.

Elite Muslim or upper class Kashmiri ladies and missionary women did not always get along. Tensions and opposition were occasionally generated by issues of modesty, purdah observance, eating habits, and religious instruction. Feminist historians such as Maina Chawla Singh acknowledge the contributions of women missionaries while noting that colonial and racial hierarchies persisted, as Kashmiri women were largely trained as assistants and nurses rather than treated as equals within medical institutions [23]. Medical encounters were venues of negotiation rather than unilateral missionary influence, as evidenced by the fact that some patients accepted biomedical therapy while rejecting overt evangelical efforts.

## Biographies of female medical missionaries and their contributions

The biographies are meant to serve as a collective illustration of larger trends in women’s missionary medicine in Kashmir, rather than just individual life sketches. When combined, they show how female medical missionaries developed institutional healthcare through hospitals and dispensaries, acquired access to female patients in a society that was gender-segregated, and integrated medical practice with evangelism and education. The profiles also show how women’s medical missions gradually became more professional and how important women’s roles as physicians, nurses, and administrators were to the growth of healthcare facilities in colonial Kashmir. When taken as a whole, these lives show how colonialism, gender, religion, and medical modernity intertwine in the area.

This study argues that women’s medical missionary work represented not merely an extension of evangelical activity but a strategic restructuring of missionary engagement in colonial India. Although earlier missionary discourse used the terms “medical missionary” and “medical women” primarily for trained female physicians (and at times nurses), the professionalization of women’s medical work in the early twentieth century marked its transformation into a defined institutional field [24].

Medical practice moved from the margins to the centre of missionary strategy. It functioned as a pragmatic entry point into communities that resisted direct evangelization. Therapeutic intervention created relational access where preaching alone failed. Thus, medicine was not auxiliary to mission; it became its operational core. The rise of biomedical authority and the formal training of women doctors enhanced both credibility and effectiveness, reinforcing the linkage between science and evangelism.

Furthermore, medical missions expanded women’s public roles within a framework that otherwise restricted female preaching. Healthcare, education, and welfare provided socially sanctioned avenues for female participation while simultaneously advancing imperial and religious agendas. India’s appeal to female recruits shaped by narratives of seclusion, cultural distance, and unmet medical need demonstrates how gendered representations of the “East” were mobilized to sustain missionary recruitment and ideology.

Women’s missionary initiatives and medical missions cannot be analytically separated, as both developed through overlapping objectives, personnel, and institutional frameworks. The expansion of women’s participation in overseas missions was closely tied to the emergence of healthcare as a structured missionary strategy. Although medical missions are often described as a comparatively late nineteenth century development frequently requiring theological justification within Protestant circles this narrative of novelty is contested. Christoffer Grundmann challenges the assumption that medical work was a modern innovation, arguing instead that healing practices have been embedded in Christian mission since its earliest phases [25]. Women’s medical missions formed part of a global network shaped by the labour of numerous committed women, many of whom remain historically overlooked. Any attempt to write about these women requires caution, as broad generalizations risk obscuring significant internal differences. Their experiences were shaped by diverse social, cultural, denominational, and professional contexts; therefore, they must be understood as a heterogeneous group rather than a uniform category [26].

While earlier scholarship often explains women’s participation in missions before 1914 as a product of limited professional options within respectable middle-class femininity, this study argues that twentieth-century women medical missionaries acted out of deliberate commitment. Many were already trained doctors or nurses who could have pursued careers in Britain or within secular medical services in India. Instead, they chose missionary medicine because they believed it offered a divinely sanctioned and socially meaningful vocation centered on Indian women.

Their work was not simply charitable outreach but a relational and negotiated engagement through which they constructed purpose and moral authority. In these interactions, missionary women understood themselves as contributing tangibly to women’s health and well-being.

At the same time, the demand for unmarried women missionaries reflected gendered realities in the sending societies. Married missionary women were often constrained by domestic and maternal responsibilities, which limited sustained professional engagement. Consequently, single women became crucial to the expansion and continuity of medical missions.

The ministry of healing needs the proclamation of the revelation of salvation in order to express its true identity: and the proclamation of salvation needs the actual concrete experience of healing, as it also seeks to make the ministry of healing possible, in order to give expression to its own intention [27].

English medical graduate Mary Brown (1864–1956) initially worked in India as the first medical missionary of the Baptist *Zenana* Mission in Ludhiana. French [28], In 1894, Edith Brown established the North India School of Medicine for Christian Women, an institution that later received affiliation with Punjab University in Chandigarh. In 1952, following the admission of male students, it was renamed Christian Medical College, Ludhiana. Recognized as the earliest institution in Asia dedicated to the formal medical education of women, it emphasized service to rural and underserved populations. Brown also attained international prominence through her participation as a delegate at the 1910 Edinburgh Missionary Conference [29]. Elizabeth Mary Browne was a loyal evangelist, a linguist and a nurse who worked as a sister at Kings College Hospital London [29], where she met Robert Clark. After marrying Robert Clark in 1858 at Marylebone Church, they embarked on a journey together to India a month later. In India, Robert Clark, the founder of the Kashmir Medical Mission [30], and his wife Mrs. Elizabeth [31], a medical specialist, played a pivotal roles in introducing the western concept of medicine in the Kashmir valley. Inspired by his wife’s success as a doctor, Robert Clark initiated the female medical mission in Jammu and Kashmir [32]. Together, they established the region’s first informal dispensary for women [33], later known as Zenana Shafa Khana, (Directorate of Health Services Kashmir) situated in Nawa Kadal, where the Diamond Jubilee hospital Neve [1, 30]. Miss Fanny Jane Butler, born on October 5, 1850 [34], known locally as “Doctor Miss Sahib,” was the first qualified British female doctor in Kashmir [32]. In 1880, she committed herself to serving women in India, initially beginning her work in Jabalpur. Motivated by strong religious ideals from an early age, she aspired to pursue missionary service and accordingly equipped herself through rigorous linguistic and professional preparation. Her decision to study medicine was shaped by the experience of caring for her ailing sister, which inspired her to adopt healing as a vocation. Upon completing her medical training, she entered missionary service in India, first working in Bhagalpur and subsequently in Kashmir. Her medical practice quickly drew large numbers of patients seeking treatment. In 1888, at the invitation of Dr. Arthur Neve, Surgeon of the Kashmir Mission Hospital in Srinagar, she arrived in the Valley and became the first formally qualified woman physician to introduce and practice allopathic medicine in Kashmir [18]. She joined the Church of England Zenana Medical Society (CEZMS).

In Bhagulpur, Miss Butler immersed herself in dispensary work, tending to the thousands of outpatients who sought the help of a medical missionary. Despite the affection and enthusiasm of the Bhagulpur [34] people. In 1888, Miss Butler was invited to Kashmir, where she collaborated with Miss Elizabeth Gordon Hull to establish a dispensary and provide medical services. In her pamphlet *Itineration in the Villages of Kashmir*, Miss Hull depicted Butler as energetic and resilient, often choosing to walk rather than rely on her restless pony while travelling through rural areas [32]. Although her health steadily declined and persistent exhaustion ultimately contributed to her premature death, Miss Butler continued her work with remarkable composure and optimism. She remained steadfast in her commitment, displaying resilience and a willingness to serve despite physical strain. In her reflections on Miss Butler’s service, Mrs. Bishop drew attention to the demanding circumstances under which she laboured, particularly at the dispensary entrance, where large gatherings of women assembled daily. The overcrowding, suffocating atmosphere, and unhygienic surroundings underscored both the intensity of the demand for medical care and the formidable conditions within which she carried out her mission [32]. Miss Butler died of typhoid fever on 26 October 1889 and was laid to rest in Srinagar. Despite her physical exhaustion, she remained spiritually composed in her final days. In a gesture of loyalty and respect, her attendants carried her body to the grave. She is remembered as an early pioneer of women’s medical missionary work in Kashmir, whose life exemplified commitment, service, and sacrifice.

She was buried in Srinagar, but her influence on women’s medical work in India continued. As an early female physician in Kashmir, she inspired future generations of women doctors. Though she did not live to see the establishment of the John Bishop Memorial Hospital in Srinagar (now relocated to Anantnag), her work laid the foundation for organized medical care for women. A scholarship was later established in her memory by the London School of Medicine for Women [7].

Miss Bessie Neve and Miss Nora played significant roles at the Kashmir Mission Hospital. Mrs. Bessie Neve, wife of Arthur Neve, was a missionary in her own right, initially working with women in Amritsar. After marrying Arthur, she transitioned to working at the Kashmir Mission Hospital [6]. Another significant contributor was Miss Neve, a relative of Dr. Arthur Neve, who reached Kashmir in 1891 and initially assisted informally at the hospital. After undergoing professional nursing training in London, she returned in 1898 as a representative of the Church Missionary Society and took charge as Superintendent of Nursing at the Kashmir Mission Hospital. Recognized for her strict discipline and emphasis on hygiene, she elevated standards of patient care and institutional order. Working with fellow medical missionaries, she helped develop the hospital from a small establishment into a more organized and efficient medical institution, extending treatment to large numbers of patients from both within and outside Kashmir.

Miss Elizabeth Mary Newman, a professionally trained nurse, was associated with the Rainawari Mission Hospital and reached Srinagar in December 1888. Alongside her clinical responsibilities, she was deeply involved in charitable and community-oriented initiatives. Her service to Kashmiri women extended beyond bedside care to include fundraising for medical institutions and support for orphaned children. In recognition of her dedicated humanitarian work, she was awarded the silver Kaiser-i-Hind medal. Tyndale Biscoe later referred to her as the “*Florence Nightingale of Kashmir*,” acknowledging her distinguished role in the region’s medical mission history.” (Directorate of Health Services Kashmir) highlighting her significant influence and dedication to healthcare in the region.

Miss Irene Verita Petrie, known affectionately as “*Niki Mem*” or the Little Lady [32] dedicated her life to serving the people of Kashmir as an exemplary medical missionary. She devoted her life to medical service in Kashmir, emerging as a central figure in missionary healthcare in the region. Through sustained clinical work and community engagement, she provided essential treatment to populations with limited access to formal medicine. Her commitment to patient care earned widespread trust and recognition across the valley. The institutional and humanitarian foundations she helped establish continued to influence healthcare practices long after her lifetime, reflecting the enduring impact of her service-oriented approach. Irene describes her first visit as, “*I waited near the gathering crowd of patients*,* who were standing and squatting in dejected attitudes in the big waiting-room till the doors opened. One old*,* feeble man with such a weary expression*,* and one poor little baby unrolled from the depths of a shapeless bundle*,* struck me particularly. Then I accompanied Miss Newnham and Miss Neve on their morning rounds in the women’s wards*,* where their gentle*,* skilful fingers are daily occupied in dressing probably several dozens of ghastly wounds. The nattiness of all the arrangements was as much to be admired as the patience of many of the sufferers. None were purdah women*,* and in many cases not only their husbands but their other relatives come to watch operations and dressings. I then saw the clean*,* well stocked dispensary*,* where groups of patients were receiving medicines*,* and eyes were being attended to; and the men’s wards*,* where the men looked up so gratefully*,* making their salaams in response to inquiries.*” [22]. In examining women’s engagement with missionary activity at the turn of the twentieth century, Jeffrey Cox refers to Irene Petrie, who served in India and died of typhoid in 1897. Her life was later commemorated in a popular biography authored by her sister. Cox suggests that her motivations were relatively clear: she sought to contribute to social and moral reform by strengthening Christian institutions in non-Christian settings. Her case raises a broader question about how many similarly motivated women participated in missionary work during this period?” [35], p. 195] Some scholars maintain that women entered missionary service out of a general desire for philanthropic engagement, viewing missions as a respectable avenue for social contribution. In contrast, Cox contends that their commitment was more explicitly religious in orientation: rather than merely pursuing benevolent work, these women consciously aligned themselves with Christian evangelistic and institutional objectives.

## Obstacles and the public’s reaction to Western medicine

In early colonial India, social norms often prevented women from consulting male physicians, which further restricted their access to formal healthcare. Colonial discourse frequently portrayed the marginal status of Indian women as evidence of societal “backwardness,” thereby reinforcing claims of imperial intervention as reformist and civilizing. Within this context, women medical missionaries occupied a critical intermediary role by providing culturally acceptable treatment to female patients. A lasting institutional outcome of medical missions was the establishment of training centres for medicine and nursing. These institutions produced some of the earliest cohorts of professionally trained Indian healthcare workers many of them women and significantly expanded women’s access to clinical care delivered by female practitioners [36]. In the nineteenth and early twentieth centuries, women medical missionaries in Kashmir worked within restrictive gender norms that limited women’s access to male doctors while simultaneously questioning female professional authority. They also faced social skepticism and regional instability, which disrupted medical continuity. Despite these challenges, they adapted to local conditions, built trust through sustained service, and contributed significantly to the development of healthcare and medical education in the region. Dr. Mary Hogarth, a medical missionary, concentrated on improving maternal and child health, thereby strengthening local confidence in institutional medicine and contributing to the early development of nursing practice in the region. Broader reformist currents in education—associated with figures such as Raja Ram Mohan Roy—also shaped the intellectual climate in which medical missions operated. Expanding educational initiatives fostered greater social awareness across religious communities and indirectly facilitated the acceptance of professional women in public service roles. Linguistic and cultural distance posed additional challenges. For example, Dr. Fanny Butler recognized language as a fundamental barrier to effective care. Rather than commencing work immediately, she postponed her mission activities until she had acquired proficiency in Kashmiri, underscoring the importance of communication in establishing medical credibility and trust [34]. News of the impending arrival of a woman doctor circulated quickly, leading many women to seek treatment at the dispensary, including the wives of artisans, boatmen, and residents from nearby villages. At the outset, Dr. Butler faced an acute shortage of trained assistants. She therefore combined clinical care with patient instruction, explaining the causes of illness and overseeing basic procedures, while major surgical cases were referred to the Neve brothers.

Public responses to Western medicine in the state were uneven. Sections of the educated elite welcomed it as evidence of administrative progress, whereas segments of society hesitated due to religious scruples, caste considerations, or circulating suspicions about new treatments. Political authority also shaped its expansion [37]. Under British paramountcy, the rulers of Jammu and Kashmir supported biomedical institutions partly to align with imperial expectations and partly to reinforce their own legitimacy. Investment in hospitals and dispensaries thus became intertwined with both governance and the projection of modern statecraft.

Indigenous medicine practitioners suffered most after western medicine became state medicine. They responded to revitalize indigenous systems. They strongly resisted the medical registration laws that expelled them from health care. All Kashmir Unani Tibbi Conference Kashmir and J and K Vedic Sabha were founded in 1937 to promote Indian medicine in the state [38]. The setting up of translation bureau and translation of indigenous medical works was part of revival movement.

Over time, biomedicine gained wider acceptance in the state, and increasing numbers of patients sought treatment in mission facilities. Missionary accounts, however, often suggest that many individuals turned to allopathic care only after indigenous remedies failed, indicating the continued strength of local therapeutic traditions. Elmslie observed that the Maharaja and sections of the elite gradually acknowledged the clinical effectiveness of Western methods.

Access also shaped patterns of use. Indigenous practitioners were embedded in rural society and readily available, whereas Western medicine was initially concentrated in urban centres. Missionaries linked healing with evangelism, viewing medical care as both therapeutic and spiritually transformative. Early adoption appears to have been stronger among socially marginalized groups, who were less constrained by orthodox hierarchies. Conversely, some pandits opposed missionary medicine and petitioned the Maharaja to restrict engagement with mission hospitals.

As biomedical treatments demonstrated visible results, resistance diminished and broader sections of society accepted them. Importantly, in Jammu and Kashmir the state did not legally compel their use; patients generally exercised choice. This contrasts with regions such as Travancore, where rulers more actively promoted and, at times, pressured populations to adopt Western medical practices [39]. Western medicine and immunization were resisted in Travancore, but the state required them for government positions. Jammu & Kashmir did not promote or denigrate allopathic medicine using these approaches. Missionary doctors spread medicine and Christianity, which alarmed religious leaders. *Mullahas*, who opposed Christianity, dispersed Dr. Elmslie’s mob at Hazratbal [22]. Priests and Brahmans resisted western doctors.

Mission records indicate that Christian missionaries were among the first to institutionalize allopathic healthcare in Kashmir and Ladakh. Inspired in part by their initiatives, Maharaja Ranbir Singh later extended similar medical arrangements across the state. Initial responses to Western medicine were ambivalent. Earlier rulers had been wary of missionary preaching, and many sections of society hesitated to accept unfamiliar therapies. Over time, however, biomedical treatment gained official backing and gradually assumed the position of a recognized state-supported system. Although missionaries did not formally displace indigenous healing traditions, their work introduced a new therapeutic framework that reshaped medical practice in the region.

Kashmir was often described as a challenging mission field where visible results required patience. Yet reports suggest that the mission’s position improved over time. Periods marked by hostility such as restrictions on hospitals and schools, harassment of converts, and seasonal withdrawal from the valley gave way to greater tolerance. The appointment of Dr. Arthur and Ernest Neve to supervise the State Leper Asylum signaled official confidence in missionary physicians. While administering clinical care, they were also permitted to communicate their religious message, linking bodily healing with spiritual instruction [40].

## Conclusion

Female medical missionaries significantly influenced the evolution of healthcare in colonial Kashmir throughout the late nineteenth and early twentieth centuries, while their contributions are sometimes neglected. Their contribution transcended philanthropic medical assistance and became important to the development of institutional healthcare for women in the region. They established new channels of medical access for Kashmiri women constrained by restricted social norms and purdah through zenana dispensaries, maternity care, nursing services, and hospital operations.

This study illustrates that missionary medicine in Kashmir operated as a multifaceted convergence of healthcare, religion, gender, and colonial authority. Missionary organizations perceived medicine as a tool for evangelism and social reform, although native women often sought care at mission hospitals and clinics for pragmatic therapeutic reasons rather than for religious conversion. Consequently, medical encounters evolved into negotiated environments influenced by accommodation, selective acceptance, and cultural interaction rather than unilateral missionary imposition.

The experiences of individuals like Elizabeth Clark, Fanny Butler, Bessie Neve, Elizabeth Newman, and Irene Petrie combined illustrate overarching trends in women’s missionary medicine. These women functioned not only as physicians and nurses but also as instructors, administrators, and facilitators between Western medicine and Kashmiri society. Their efforts facilitated the progressive professionalization of women’s healthcare and enhanced women’s involvement in public medical domains.

This study concurrently underscores the limitations and paradoxes inherent in missionary medicine. Missionary criticisms of indigenous healers and dais embodied colonial preconceptions regarding local medical practices; nonetheless, indigenous systems persisted alongside biomedical institutions within a pluralistic medical landscape. Kashmiri women were not merely passive recipients of Western medicine; rather, they were active participants who negotiated medical decisions in accordance with their cultural and social contexts. The history of women medical missionaries in Kashmir illustrates how gendered medical labor influenced the overall advancement of healthcare, public health involvement, and medical modernism in the region.

## Data Availability

No datasets were generated or analysed during the current study.
